# Supplementary material for: Reconciling Mining with the Conservation of Cave Biodiversity: A Quantitative Baseline to Help Establish Conservation Priorities
Source: PLoS One. 2016 Dec 20;11(12):e0168348. doi: 10.1371/journal.pone.0168348 (PMC5173368; doi:10.1371/journal.pone.0168348)
Supplement: S1 Dataset — (ZIP) [file pone.0168348.s002.zip › Taxa/Serra Norte/SN_2013/N5S/Lista N5S-62.pdf]

CAVIDADE N5S-0062

Classe	Ordem	Fam/Outros	Gên/Outros	Espécie	S-U	U-S	Zon.
Arachnida	Acari			jovem	X		P
Arachnida	Amblypygi	Charinidae	*Charinus*	sp.1		1	P
Arachnida	Amblypygi	Phrynidae	*Heterophrynus*	*longicornis*	5	4	E/P
Arachnida	Araneae	Caponiidae		jovem	X	X	E/P
Arachnida	Araneae	Drymusidae	*Drymusa*	*spelunca*	X		E
Arachnida	Araneae	Ochyroceratidae	*Speocera*	sp.1	X	X	P
Arachnida	Araneae	Palpimanidae		jovem	X		E
Arachnida	Araneae	Pholcidae	*Mesabolivar*	*aurantiacus*	X	X	E
Arachnida	Araneae	Salticidae		jovem	X		E
Arachnida	Araneae	Theridiidae	*Theridion*	sp.4	X	X	P
Arachnida	Araneae	Theridiosomatidae	*Plato*	sp.1	X		P
Arachnida	Opiliones	Cosmetidae	*Discosomaticinae*	sp.1	X		E
Arachnida	Opiliones	Escadabiidae		sp.2		X	P
Arachnida	Pseudoscorpiones	Chthoniidae	*Pseudochthonius*	sp.1	X		P
Diplopoda	Glomeridesmida	Glomeridesmidae		sp.1		1	P
Diplopoda	Polydesmida	Fuhrmannodesmidae		jovem	X		E
Diplopoda	Polydesmida	Pyrgodesmidae		sp.1	X	X	P
Entognatha	Collembola	Cyphoderidae		sp.1		X	E
Entognatha	Collembola	Paronellidae		sp.1		X	E
Entognatha	Diplura	Campodeidae		sp.1		X	E
Entognatha	Diplura	Projapygidae		sp.1	X		E
Insecta	Blattodea	Blattidae		sp.4		1	E
Insecta	Coleoptera	Carabidae		sp.3		X	P
Insecta	Coleoptera	Dytiscidae		sp.1		X	P
Insecta	Coleoptera	Dytiscidae		sp.6		X	E
Insecta	Coleoptera	Hydrophilidae		sp.1		X	P
Insecta	Diptera	Tipulidae	Tipulinae	sp.	X	X	E/P
Insecta	Heteroptera	Hebridae	*Hebrus*	jovem	X	X	E/P
Insecta	Heteroptera	Pentatomoidea		jovem	X		E
Insecta	Heteroptera	Reduviidae	Emesinae	sp.1	1	1	E/P
Insecta	Homoptera	Cixiidae		jovem	X	X	E/P
Insecta	Hymenoptera	Formicidae	*Brachymyrmex*	sp.1	X	X	E
Insecta	Hymenoptera	Formicidae	*Hypoponera*	sp.1	X	X	P
Insecta	Hymenoptera	Formicidae	*Paratrechina*	sp.1	X		E/P
Insecta	Hymenoptera	Formicidae	*Pheidole*	sp.3		X	E
Insecta	Hymenoptera	Formicidae	*Trachymyrmex*	sp.1	X		P
Insecta	Isoptera			sp.	X	X	E
Insecta	Lepidoptera	Noctuoidea		sp.5	1		P
Insecta	Orthoptera	Phalangopsidae	*Paraclodes*	sp.	2	2	E
Insecta	Orthoptera	Phalangopsidae	*Phalangopsis*	sp.	9	19	E/P
Gastropoda	Pulmonata	Systrophiidae	*Happia*	sp.	X		P
Amphibia	Anura			sp.	3		E
